# Study of the Long-Term Aging of Polypropylene-Made Disposable Surgical Masks and Filtering Facepiece Respirators

**DOI:** 10.3390/polym15041001

**Published:** 2023-02-17

**Authors:** Sandra Carreiras-Suárez, Lidia Domínguez-Ramos, Massimo Lazzari

**Affiliations:** 1 Departamento de Química Física, Facultade de Química, Universidade de Santiago de Compostela, 15782 Santiago de Compostela, Spain; 2 Centro Singular de Investigación en Química Biolóxica e Materiais Moleculares (CiQUS), Universidade de Santiago de Compostela, 15782 Santiago de Compostela, Spain; 3 Departamento de Ingeniería Química, ETSE, Universidade de Santiago de Compostela, 15782 Santiago de Compostela, Spain

**Keywords:** weathering, photoaging, microplastics, polymer oxidation, ATR-FTIR spectroscopy

## Abstract

The main purpose of this work is to contribute to understanding the mechanism of oxidation of the polymeric components of common disposable masks used during the COVID-19 pandemic to offer the chemical basis to understand their long-term behavior under typical environmental conditions. Artificial aging of representative mask layers under isothermal conditions (110 °C) or accelerated photoaging showed that all the PP-made components underwent a fast oxidation process, following the typical hydrocarbon oxidation mechanism. In particular, yellowing and the melting temperature drop are early indicators of their diffusion-limited oxidation. Morphology changes also induced a loss of mechanical properties, observable as embrittlement of the fabric fibers. Results were validated through preliminary outdoor aging of masks, which allows us to predict they will suffer fast and extensive oxidation only in the case of contemporary exposure to sunlight and relatively high environmental temperature, leading to their extensive breakdown in the form of microfiber fragments, i.e., microplastics.

## 1. Introduction

Surgical masks and filtering facepiece respirators are personal protective equipment used to protect the wearer from particles or liquids contaminating the face. They have become a global requirement in the past two years to limit SARS-CoV-2 diffusion as one of the precautionary measures to slow down the transmission rate of coronavirus disease 2019 (COVID-19) [1]. Consequently, their production has increased at an unprecedented rate since the middle of 2020 [2] to cover the need for around 130 billion disposable masks per month [3].

The materials used for the manufacture of face masks are fundamentally synthetic thermoplastic polymers, polypropylene (PP) being the most commonly used [1], so the increase in their consumption is undoubtedly contributing to the already alarming problem of plastic pollution in the environment [2]. Due to their long-term stability and durability, which may be estimated in the order of tens to hundreds of years, plastics tend to accumulate in the environment [4]. In particular, it is estimated that of the total plastic generated since its large-scale production began in the 1950s, 75–80% (corresponding to more than 5000 metric tons) has been dumped in landfills or permeated into the natural environment [5].

Single-use polymeric materials are considered one the main sources of microplastics (particles with a diameter of less than 5 mm) since they may degrade and fragment under environmental conditions. These microplastics threaten terrestrial and marine ecosystems, global food security, and the tourism industry and exacerbate climate change and the spread of pathogens [2]. Although the weathering and the degradation under different conditions of the most important classes of industrial polymers (including PP) were extensively studied [6,7,8,9,10], their aging behavior in the form of nonwoven fabrics, such as those used for the manufacture of disposable face masks, has been investigated mostly in general terms, essentially focusing on field observations and within recycling proposals [2,11,12,13,14,15]. Some studies highlight the risk of microplastic release from both surgical masks and FFP2 filtering facepiece respirators due to fragmentation processes, e.g., [2,12,16,17], but without focusing on the microplastic formation mechanism, especially after weathering and other aging processes.

The main goal of this work is to contribute to the understanding of the mechanism of oxidation of the polymeric components of the most common disposable masks used during the COVID-19 pandemic to offer the chemical basis to predict their long-term behavior under typical environmental conditions, i.e., in the presence of oxygen at moderate temperatures. Special attention was paid to explaining the processes entailing the release of small fragments. The oxidative degradation behavior of the different layers of representative commercial masks was followed under accelerated degradation conditions. Polymer structural and morphological changes were followed during degradation by attenuated total reflectance Fourier transform infrared (ATR-FTIR) spectroscopy and differential scanning calorimetry (DSC), while the formation of volatile compounds and surface changes were monitored by gravimetric determination, optical microscopy, scanning electron microscopy (SEM), and color measurement. A first preliminary validation of the results of the accelerated aging treatment was also carried out by direct comparison with the behavior of surgical masks exposed to natural aging conditions.

## 2. Materials and Methods

### 2.1. Materials

Three commercially available disposable masks, selected among those available in Spain, were labeled as follows: type IIR certified surgical masks model A (Carrefour Soft, 10 masks/pack, Tarragona, Spain) and model B (Mask4u PI Medical labs, 10 masks/pack, Badajoz, Spain), and type FFP2 certified filtering respirator model C (Mooncare FFP2 YD-002, 5 masks/pack, Dongguang, China). All the items report a CE mark, indicating that they respect the EU Personal Protective Equipment Regulation 2016/425. The final choice of the selected masks did not follow any commercial interest but was simply reflecting the most typical typologies, as detailed in the results section.

### 2.2. Characterization Techniques

IR absorption spectra in ATR mode were collected with a Thermo Nicolet 6700 FTIR instrument equipped with a Smart Endurance device and a mercury cadmium telluride (MCT) detector, liquid nitrogen cooled (spectral range 4000–670 cm^−1^), at 4 cm^−1^ resolution for 128 scans. Carbonyl indexes were normalized against the absorption at around 1455 cm^−1^ ascribed to the CH_2_ bending vibration of PP and considered approximately constant during the early stages of oxidation. Data were processed with Omnic 8.1 by Thermo Nicolet.

DSC thermograms were obtained with a Q200 (TA Instruments, New Castle, DE, USA) calorimeter equipped with a refrigerated cooling system in the temperature range from −70 to 200 °C, using 3–5 mg samples with a scanning rate of 20 °C min^−1^, under a 50 mL min^−1^ nitrogen flow. Weight changes were determined gravimetrically with an AND Gr-200 analytical balance with a 0.0001 g repeatability and ±0.0002 g linearity, using 15–20 mg samples. Optical microscopy images were obtained using a Dino-Lite AM7915MZT digital microscope (AnMo electronics, New Taipei City, Taiwan. Morphological and structural features of the samples were also assessed by field emission SEM after sputter coating with a 5 nm Ir layer. The images were obtained using a Zeiss FESEM Ultra Plus (Oberkochen, Germany), working at 3 kV, on three different areas per sample, using at least three different magnifications. The diameters of the fibers are reported as a range of values referring to about 90% of the fibers.

Color measurements were performed with a portable Konica Minolta CM 700d spectrophotometer (Konica Minolta, INC., Tokyo, Japan). Color space was the CIELAB with D65 standard illuminant and 10° observer, ø 6 mm illumination area. The International Commission on Illumination (CIE) developed this L*a*b* color model in 1976 to measure objective color and calculate color differences. The letters L*, a*, and b* represent each of the three coordinates of the 3D color space. L* represents lightness from black to white on a scale of 0 to 100, while a* and b* represent chromaticity with no specific numeric limits, indicating color position between red and green (where negative values indicate green and positive values indicate red) and between yellow and blue (where negative values indicate blue and positive values indicate yellow), respectively. SpectraMagic NX software was used. The reported coordinates refer to the diffuse reflectance spectra (specular component excluded). Color change, $\Delta E_{12}^{*}$, representing the distance between two given points in the 3D color space, with coordinates $L_{1}^{*}$, $a_{1}^{*}$, and $b_{1}^{*}$ and $L_{2}^{*}$, $a_{2}^{*}$, and $b_{2}^{*}$, respectively, is defined as follows:

(1) $$\Delta E_{12}^{*}=\sqrt[{2}]{{\left(L_{2}^{*}-L_{1}^{*}\right)}^{2}+{\left(a_{2}^{*}-a_{1}^{*}\right)}^{2}+{\left(b_{2}^{*}-b_{1}^{*}\right)}^{2}}$$

### 2.3. Aging Treatments

Isothermal treatment at a temperature of 110 °C was performed in a static oven. Accelerated photoaging treatments were carried out in a Suntest CPS+ or XLS+ (both Heraeus) high-speed exposure unit equipped with a xenon light source with constant irradiation at a power of 765 W/m^2^. A glass filter with a cut-off at λ less than 295 nm was used to exclude radiation more energetic than that of outdoor daylight exposure. The maximum temperature of the samples during irradiation was 24 and 44 °C black panel temperature in the XLS+ and CPS+ units, respectively, as maintained by a forced-air circulation system.

Model A and B masks were also submitted to outdoor weathering. Masks without disassembling were fixed with tape onto a red ceramic external floor exposed to Atlantic oceanic weather for 82 days between April and June 2021 (La Coruña, Spain; latitude: 43.382763 WGS84, longitude: −8.409202 WGS84-EPSG:4326). Weather data were as follows: maximum ambient temperature 24.7 °C, minimum ambient temperature 6.4 °C, average ambient temperature 15.7 °C, average relative humidity 82%, total rainfall 266 mm, average solar irradiation 10 KJ/m^2^·day, temperature of the red ceramic surface 4–42 °C.

## 3. Results

### 3.1. Material Characterization

Two types of disposable masks were selected among those commonly used for personal protection from airborne diseases, especially during the COVID-19 pandemic, i.e., surgical masks (type IIR certified) and filtering respirators (type FFP2 certified). Identification of the polymeric components of the fabric layers was carried out by ATR-FTIR spectroscopy based on the assignment of the main characteristic vibration bands [15] and through optical microscopy to discriminate between manufacturing processes. All the results shown in this work refer to only three specific commercial items. However, very similar characteristics were disclosed from the preliminary evaluation of more than 20 identical-type masks and respirators available in the Spanish market and, by extension, in the European Union market. In particular, we verified that surgical masks contain three layers of nonwoven isotactic PP fabric produced either by the spun-bonding process or melt blowing [18]. The latter consists of a softer and bulkier web with a smaller fiber diameter and pore size. Indeed, the melt-blown PP fabric constitutes the inner filter, whereas the external spunbonded layers may be different (colored outward side and white, uncolored inward side) or be uncolored and very similar to each other, as in the selected models A and B, respectively (Table 1). The dye used to fabricate the outer side of model A (layer A1) could not be identified. However, it may be supposed that a common anthraquinone or phthalocyanine blue-based master batch was mixed with PP chips by a melt dyeing process to obtain a homogeneous mass coloring [19].

On the other hand, the typical FFP2 respirator is formed by at least five layers, which in the case of the selected item, here named model C, were identified as shown in Table 1. The two external layers, C1 and C5, made of melt-blown PP, are almost identical to those found in IIR masks; C3 and C4 are made of spunbonded PP, as for the inner layer of models A and B; and C2 was identified as a PE/PET sheath–core bicomponent spunbonded nonwoven fabric (C2 ATR-FTIR spectrum is shown in Figure S1a). All the PP components of model C, as well as those of models A and B, showed almost identical spectra, except the layers C3 and C4, in which an additional peak centered at 1541 cm^−1^ is visible and can be assigned to triazinic compounds such as those belonging to hindered amine light stabilizers (C3 ATR-FTIR spectrum is shown in Figure S1b) [20]. Finally, it is worth stating that in some of the analyzed commercial FFP2 respirators, all the inner layers are made of PP, and for this reason, they were not considered within this study as not adding further variables.

On such basis, the following layers were considered as representative of the variety of materials found in masks and respirators and were submitted to artificial aging treatments:

- A1 (spunbonded light blue PP);
- A2 (melt-blown PP, identical to B2);
- B1 (spunbonded PP, very similar to A3, B3, and C5);
- C1 (spunbonded PP, very similar to B1 but with a higher fiber density);
- C2 (PE/PET hot air cotton);
- C3 (spunbonded PP with light stabilizers, as in C4).

To study the weathering of these materials in times shorter than those necessary under natural conditions, we applied a common material science practice in which the factors affecting polymer degradation are accelerated and controlled through opportune environmental chambers [21,22]. In this investigation, aging was accelerated in two different ways to compare and bolster the results: irradiation of the fabric samples in a photoaging device equipped with a solar-like lamp (filtered for ≤295 nm) or through isothermal treatments in an oven at temperatures that do not imply a regime of macromolecular motion different from that occurring under natural conditions. For PP, that corresponds to a temperature necessarily higher than room temperature but lower than its melting temperature (ca. 160 °C). It is largely accepted that the conditions of accelerated aging assays do not trigger unexpected reactions: either artificial solar light or isothermal treatment at a moderate temperature essentially accelerates the same chemical changes as those occurring in the long term under environmental conditions, also taking into account that weathering pathways for vinyl polymers are commonly related to oxidative degradation [7,22].

### 3.2. Accelerated Degradation

The temperature used for the isothermal treatment, i.e., 110 °C, was selected based on the results of preliminary tests at lower temperatures, which showed only minor structural or optical changes after exposure for up to 500 h. The choice was also taken considering previously published studies on PP oxidation, either in bulk or as thin films, emphasizing that higher temperatures are incompatible with diffusion-limited oxidation and trigger unwanted degradation processes [23,24]. Standard accelerated photoaging was carried out at a controlled temperature of 24 °C and, additionally, at 44 °C to evaluate the further effect of temperature increase on the photooxidative behavior. Formation of volatile compounds and surface changes, often associated with transformations in polymers, were monitored by gravimetric determination, SEM, and evaluation of color changes to follow the progress of degradation reactions. Moreover, ATR-FTIR spectroscopy and DSC were used to periodically check treatment-induced structural and morphological changes.

**Figure 1:**
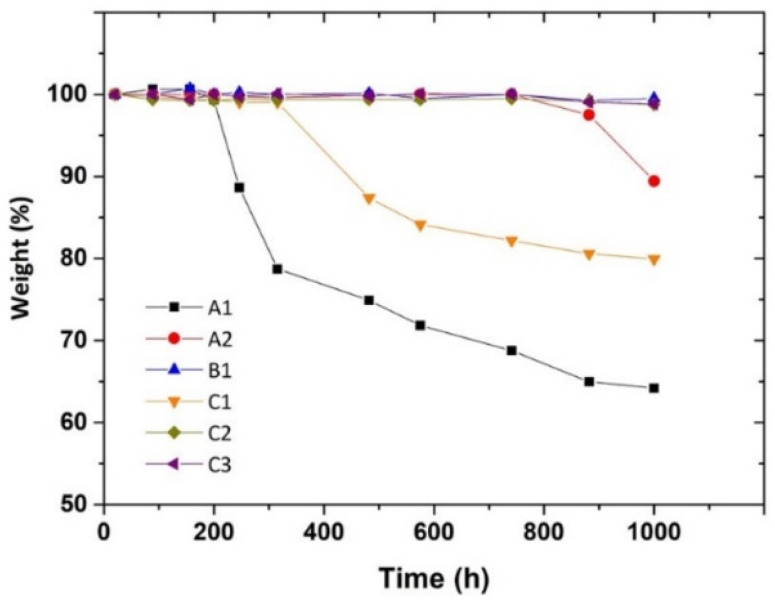
Weight changes of the layers exposed to isothermal aging at 110 °C as a function of treatment time.

**Figure 2:**
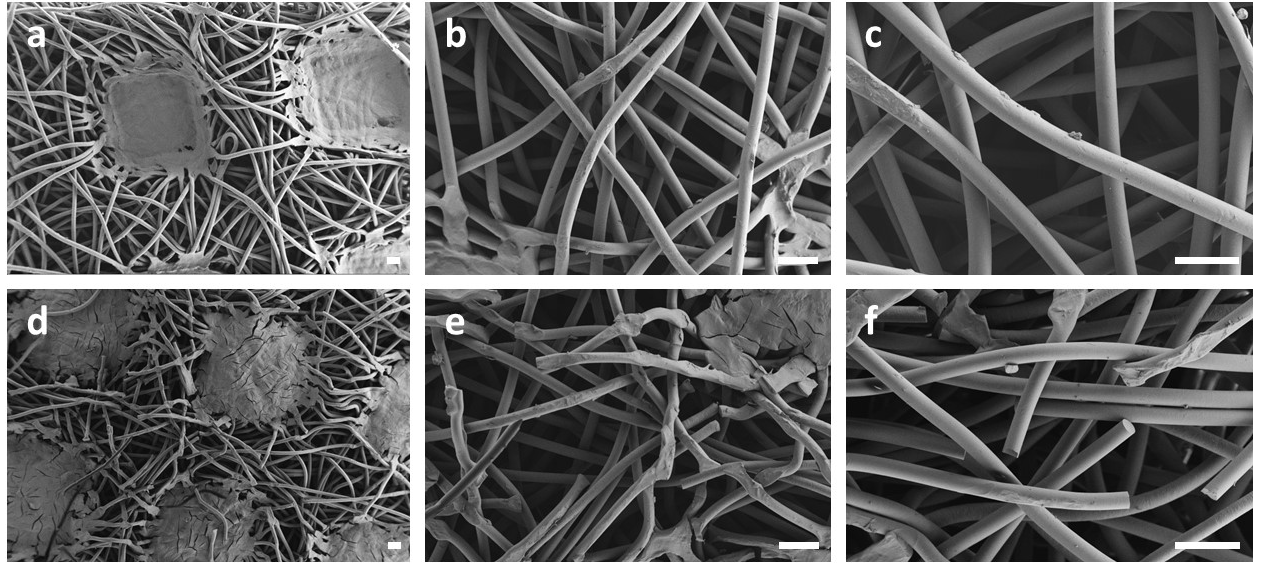
SEM images of C1 before (**a**–**c**) and after 1000 h isothermal treatment at 110 °C (**d**–**f**). Scale bar 100 μm.

The weight losses of reference specimens during the isothermal treatment were almost negligible for all the layers, except for A1, A2, and C1, which start to lose a significant weight fraction under isothermal conditions after ca. 200 h, 900 h, and 300 h, respectively (Figure 1). These data apparently contradict previous studies, indicating that a very small amount of volatile products is released by the oxidation of PP (essentially very low molecular weight oxygen-containing molecules and hydrocarbons), even at higher temperatures or under different conditions of oxidative degradation [23,25,26]. The formation of volatiles from PP layers at 110 °C cannot be excluded. However, most of the weight loss is possibly related to a different phenomenon that could be appreciated by optical microscopy and SEM, as well as through an observed increasing fragility of samples during manipulation, e.g., with pliers. Extensive rupture of fibers with a diameter of around 25 μm is especially evident in A1 and C1 (as an example, SEM images of C1 exposed to the isothermal treatment are shown in Figure 2), where a fragilization of the network leads to the formation of small fragments that eventually are released during the weighting procedure. No broken fibers were visible in the samples before aging, while this detached fraction was macroscopically perceived as impalpable dust (Figure S2) and is responsible for the weight decrease.

To support this hypothesis, it is worth highlighting that extensive embrittlement and progressive pulverization were observed after 150 h and 100 h photoaging at 24 °C and 44 °C, respectively, for layer A2 and after 325–500 h photoaging at 44 °C for the other PP layers, entailing continuous mechanical stress of the fibers exposed to the forced-air circulation. That behavior possibly indicates the beginning of the oxidation process leading to the formation of surface cracks [27], an effect which is more important for the smaller average diameter of the A2 fibers (5–20 μm) resulting from the melt-blowing processing of this specific layer. Bigger spunbonded fibers, such as those of A1 and C1, although already affected by oxidation processes (as also confirmed below by FTIR spectroscopy), do not reach critical conditions under forced-air circulation stress (at 24 °C) or do reach them at times longer than A2 fibers (at 44 °C).

**Figure 3:**
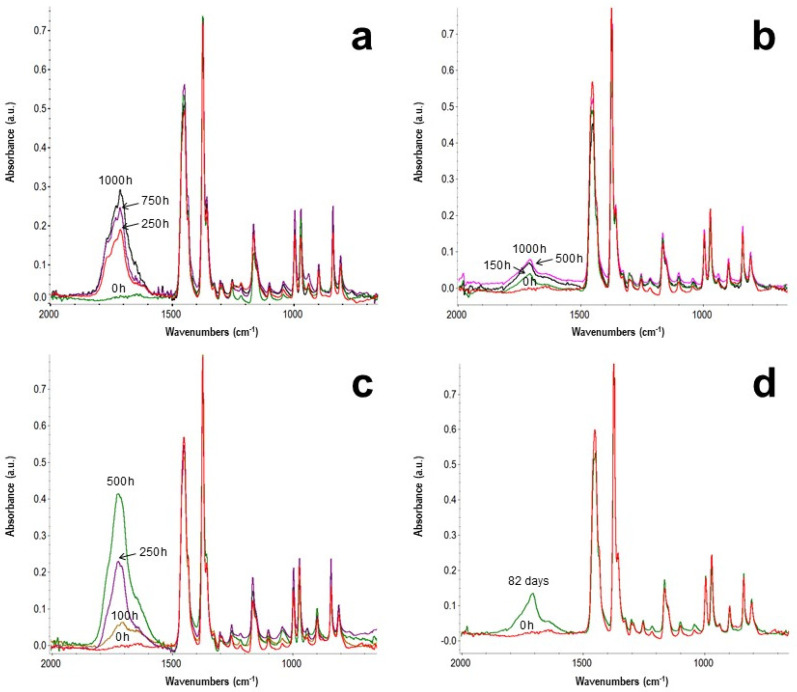
Selected ATR-FTIR spectra in the range 2000–670 cm^−1^ of A1 exposed to isothermal aging at 110 °C (**a**), accelerated photoaging at 24 °C (**b**), accelerated photoaging at 44 °C (**c**), and outdoor aging (**d**).

The evolution of structural changes shown by ATR-FTIR spectroscopy is comparable for all the PP layers under the different accelerated aging conditions and similar to that previously reported for PP films [9,28,29,30]. Only C2 did not show any relevant changes over aging, apart from a very limited broadening of the carbonyl peak, confirming the good oxidative stability of PET-based fibers. PP oxidation mainly resulted in the formation of carbonyl groups easily detectable in the 1600–1800 cm^−1^ range (as an example, ATR-FTIR spectra of A1 under isothermal and photoaging conditions are shown in Figure 3). However, distinct layers showed different oxidative extents during the considered treatment time.

**Figure 4:**
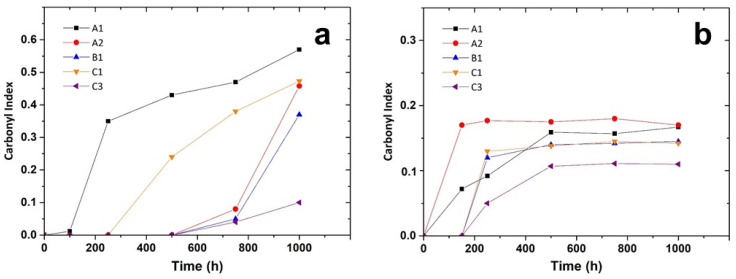
Carbonyl index from FTIR spectroscopy measurements of PP layers exposed to isothermal aging at 110 °C (**a**) and accelerated photoaging at 24 °C (**b**) as a function of treatment time.

As visible in Figure 4a, showing the carbonyl index evolution of PP layers under isothermal conditions, A1 has the shortest induction period, defined as time to carbonyl index increase onset, followed by C1. In contrast, the remaining layers show signs of oxidation only after at least 500 h of treatment. The oxidative stability order C3 > B1 > A2 > C1 > A1 is in good agreement with the abovementioned fragilization and also with the color change trends shown below, which may be tentatively ascribed to the presence of polymeric antioxidants or previous thermal processing. In the case of C3, the highest stability is due to the detected triazinic compounds acting as a stabilizer. In addition, the relative stability order of the other layers may be related to the possible presence of different amounts of stabilizers in their formulation, e.g., hindered phenolic antioxidants, typically found in PPs and difficult to be detected by FTIR spectroscopy. In addition, the highest oxidizability of A1 was related to the spun-bonding processing of PP with the blue masterbatch, obtained by thermal processing of a PP/dye mixture, which becomes more sensitive to further degradation treatments as appreciable by the absence of a relevant induction time.

On the other hand, the induction periods under photoaging at 24 °C are shorter (Figure 4b), and the carbonyl indexes rapidly reach similar plateau values, i.e., around 0.14–0.17, for all the PP layers except the triazinic stabilized C3, which are in any case smaller than those measured during the isothermal treatment. Further experiments of accelerated photoaging at 44 °C showed even shorter induction periods than those at 24 °C but with higher carbonyl peaks, with maximum carbonyl index values of around 0.45 (see, e.g., ATR-FTIR spectra of A1 in Figure 3c), and extensive embrittlement and pulverization of the PP layers that limited the treatment to 100–500 h, as abovementioned.

The achievement of different carbonyl index thresholds, depending on the accelerated aging conditions, does not imply the development of distinct degradation mechanisms but confirms that the oxidation is diffusion-limited [23,31,32]. Photoaging seems to favor the initiation of the oxidation process for isothermal conditions, but at 24 °C, oxygen starvation prevents the extensive formation of oxygen-containing groups. In contrast, their easier and deeper development at 44 °C, as an effect of higher oxygen diffusion, shortly leads to a collapse of the fabric, even under moderate mechanical stress due to the air-cooling system.

**Scheme 1:**
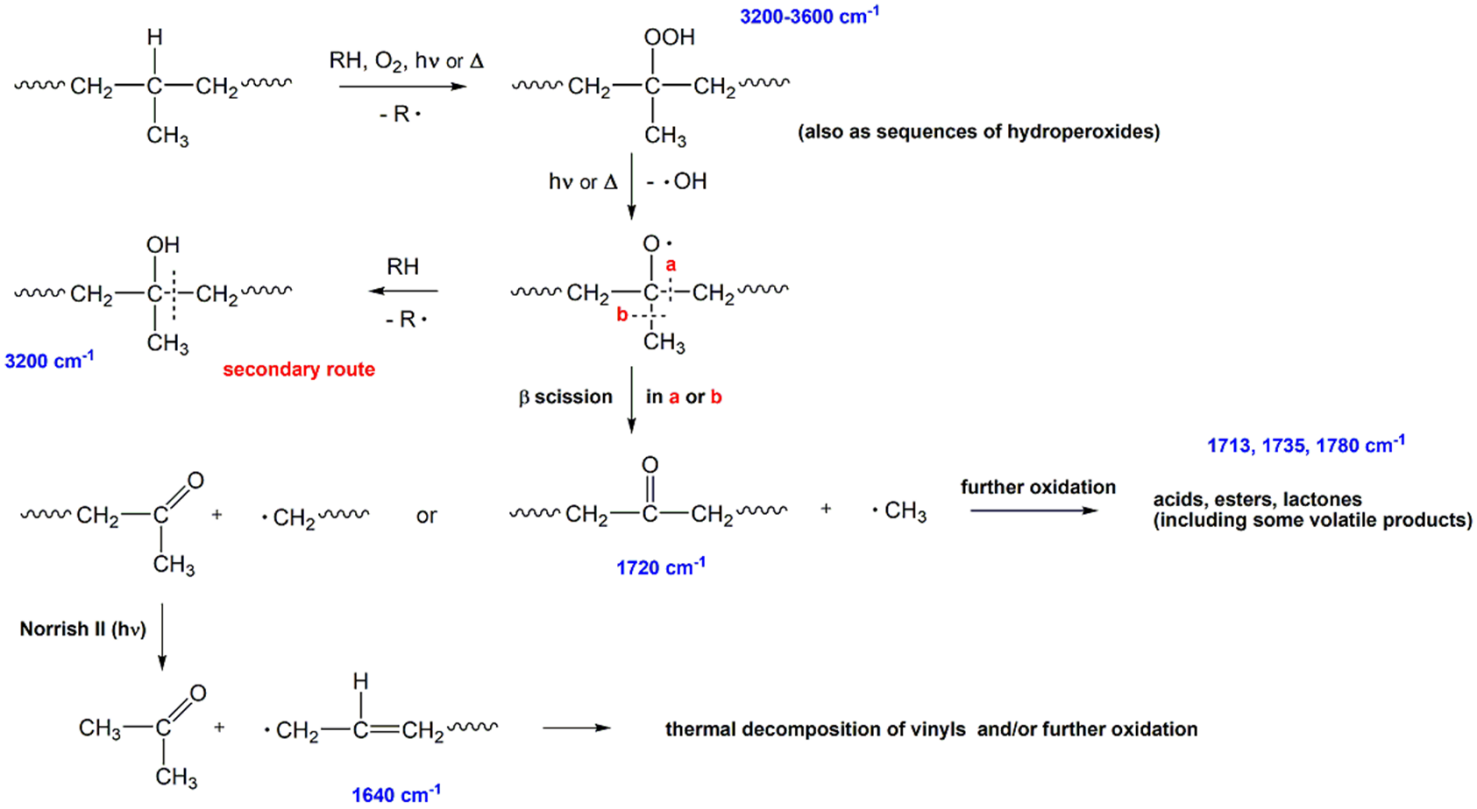
Main route of oxidation of PP, with the indication of the absorption bands of the main degradation products in FTIR spectra [27,36]. RH: polymer molecule; R: polymer radical; hν: light; Δ: heat.

Moreover, the differences between the spectra of isothermally aged and photoaged PP (such as those visible comparing Figure 3a and Figure 3b), and in particular, the presence of a clearer shoulder at ca. 1640 cm^−1^ of the carbonyl group of carboxylic acids at 1713 cm^−1^ and a broad band in the 3200–3600 cm^−1^ hydroxyl region appearance under photoaging, may be explained within a common oxidation mechanism. Photooxidation and thermooxidation of PP produce the same oxidation products, but their relative concentrations are different as an effect of photochemical Norrish-type reactions [33]. The well-known auto-accelerating mechanism of oxidation of hydrocarbons induces the formation of hydroperoxides [34,35], which decompose to form alkoxy radicals, mostly undergoing β-scission with the formation of different carbonyl compounds (Scheme 1) [36]. These degradation products are carboxylic acids (the most abundant), ketones, esters, and lactones, visible at 1713 cm^−1^, around 1720 cm^−1^, 1735 cm^−1^, and 1780 cm^−1^, respectively. Concerning the hydroxyl domain, it is agreed that the broad band peaking up between 3300 and 3500 cm^−1^ is due to the absorptions of bonded hydroperoxides with a very small contribution to the OH absorption of isolated acids. The appearance of a peak at 1640 cm^−1^ under photoaging conditions was attributed to the partial decomposition of ketones and especially acid groups by Norrish-type II reactions resulting in the formation of vinyl groups [27]. In addition, one can expect that such vinyl groups disappear as an effect of further oxidation, e.g., through hydrogen abstraction from the carbon in α-position to the C=C, but, as already observed for polyethylene [33], such a reaction is strongly affected by the temperature of photoaging. Thus, it is slower at 24 °C than 44 °C, leading to a higher accumulation of vinyl groups at the lowest temperature.

**Figure 5:**
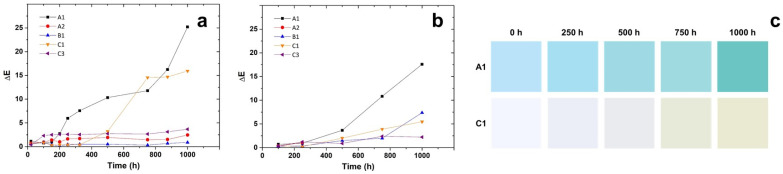
Color changes of PP layers exposed to isothermal aging at 110 °C (**a**) and accelerated photoaging at 24 °C (**b**) as a function of treatment time, and an example of the chromatic evolution of layers A1 and C1 exposed to isothermal aging at 110 °C (**c**).

Color changes of PP layers were measured using a spectrophotometer in the CIELAB color space. They were considered an early indicator of oxidation processes, also leading to the formation of chromophores. A2, B1, and C3 showed little changes during the isothermal treatment, smaller than or close to *ΔE* = 2 and 4 (Figure 5), which are considered threshold values of minimal detectable difference for saturated and unsaturated colors, respectively [37]. C1 did not show relevant changes after more than 500 h of treatment, whereas A1 color changes were clearly perceptible after 250 h, reaching *ΔE* = 25.2 at 1000 h. Images corresponding to the absolute color coordinates of some layers are shown in Figure 5c to visualize the chromatic evolution as an effect of aging directly. A more detailed analysis of the evolution of L*, a*, and b* coordinates, shown in Table S1, indicates that in all the layers, except A1, the most relevant variation is that of Δb*, indicating a progressive yellowing, reaching a maximum value of 15.5 for C1. In the case of the light blue layer A1, the color change is more complex, also indicating a progressive decrease in lightness (ΔL* = −13.4) and a conversion to greener values (Δa* = −19.3) after 1000 h, possibly due to both chromophore formation in the polymer (as in the other layers) and dye fading [38].

Accelerated photoaging at 24 °C also induces color changes but with a different effect on colorimetric parameters (Figure 5b and Table S2). Changes are generally smaller than those visible under isothermal conditions and mostly result from an extensive lightness decrease, e.g., ΔL* up to 17.5 and 7.2 for A1 and B1, respectively. In contrast, only very little changes (in the range of 0.5–1.8) were detected for the chromatic coordinates a* and b*. As a fact, layer yellowing is visually undetectable.

Furthermore, the abovementioned photochemical processes involving the decomposition of carbonyl-containing chromophores, responsible for extensive yellowing visible after isothermal aging, may account for the limited color changes detected for the chromatic coordinates a and b for the case of photoaged PP.

Advancement of degradation processes in PP may additionally be followed through an indirect evaluation of the chemical changes influencing polymer crystallinity [39]. It is well known that a typical DSC curve of PP displays a wide and complex endothermic melting peak, with a main maximum accompanied by one or more shoulders. All the PP layers showed an initial melting temperature, *T_m_*, with the maximum of the main peak in the approximate range of 155–165 °C, as expected for industrial isotactic PP. The small deviation between the layers is related to different processing parameters, e.g., melt-blown vs. spunbonded, processing temperature, or fiber diameter, which affect the final polymer molecular weight and crystallite characteristics [40]. Recent studies on the oxidation of PP films revealed the correlation between their thermal and mechanical properties and hydroperoxide formation, and in particular, the effect of the very beginning of oxidation on the *T_m_* and the crystallinity content (χ_c_) [39,41]. As visible in Table 2, where the main melting peak temperature and χ_c_ are shown, also in the case of the analyzed nonwoven fabrics, the changes in both parameters resulted as a good indicator of incipient oxidation. For example, DSC curves of A1 before and after 1000 h isothermal treatment at 110 °C are shown in Figure S3. Comparing the growth of carbonyl indexes shown in Figure 4 with *T_m_* values, a direct relationship between the appearance of carbonyls and the *T_m_* changes may be observed. In particular, aging under isothermal conditions induced a significant *T_m_* decrease in A1 and C1 after 250 h and 500 h, respectively, corresponding to their carbonyl accumulation. The shorter induction periods under accelerated photoaging at 24 °C correspond to faster *T_m_* changes (Table 3). In contrast, lower plateau values of the carbonyl indexes are associated with smaller decreases in all the layers. In addition, the values of χ_c_, calculated using a standard heat of melting for a 100% crystalline isotactic PP, *ΔH* = 209 J/g [39], confirmed the typical trend of PP oxidation, with a limited increase in crystallinity at the very beginning of oxidation, followed by a possible decrease [39,41].

This behavior seems to confirm the hypothesis of an implication of the crystallite interfaces in the initiation step of the oxidation process [39,42], where the *T_m_* drop was explained in terms of an increase in the surface energy of the crystallites as a result of the development of oxidation products on their surfaces. At the same time, such changes justify the reported sudden loss of mechanical properties of PP films before the end of the induction period [43]. They may be considered the cause of the abovementioned embrittlement of layers. Finally, as a secondary effect of PP photooxidation, it is worth remembering the so-called partial chemi-crystallization [44], which has not been specifically studied for the selected specimens but could account for the lightness decrease (Figure 5 and Tables S1 and S2) as a consequence of some changes in the PP crystalline structure.

### 3.3. Preliminary Outdoor Aging and Validation of Artificial Aging

A practical, although preliminary, validation of the correspondence between natural weathering and accelerated aging conditions was obtained through a preliminary evaluation of the behavior of surgical mask models A and B submitted side up, without disassembling (i.e., with upper layers A1 and B1 directly exposed to sunlight and layers A3 and B3 side down), to oceanic climate for around 2000 h (83 days) under a solar dosage of approximately 10 KJ/m^2^·day, with specimen surface temperature in the range of 7–40 °C. However, minor color changes were detected for all the layers (*ΔE* < 1), and ATR-FTIR spectra revealed structural changes almost identical to those obtained after shorter times of accelerated isothermal treatment or photoaging at 44 °C, pointing out an initiation of the oxidation process. Surprisingly, all the layers showed similar weathering effects, regardless of direct exposure, possibly indicating a high sunlight penetration through the different layers. For example, layers A1 and A2 showed a level of oxidation between the levels obtained in the 100–250 h range of photoaging at 44 °C, with carbonyl index values of around 0.22 for both layers (ATR-FTIR spectra of A1 in Figure 3d). The relatively less intense vinyl group shoulder at around 1640 cm^−1^, e.g., in comparison with the accelerated photoaging at 24 °C, seems to indicate that the external temperature plays an important role in addressing the whole mechanism of degradation under outdoor aging. Actually, temperatures as high as 42 °C, due to the effect of solar radiation on the ceramic support, prevent the accumulation of vinyl groups, as already discussed when comparing the two accelerated photoaging treatments.

Further speculation based on the accelerating factor, which may be determined by comparing accelerated aging treatments with natural aging, leads us to suppose a fast fragmentation of masks exposed to natural weathering. Approximately, as a time of 100–500 h of photoaging at 44 °C (depending on the PP layer) induced extensive embrittlement, the estimation of an accelerating factor of around 10 from the values of the carbonyl indexes measured, e.g., for the A1 layer after photoaging at 44 °C and under natural conditions, respectively, it is reasonable to predict a progressive fragmentation of the different PP fabrics of the masks starting from just 1000–5000 h (approximately 40–200 days) of exposure to an oceanic climate.

## 4. Conclusions

Artificial aging of representative layers of surgical masks and filtering respirators, either under isothermal conditions (110 °C) or accelerated photoaging, showed that all the PP-made components underwent a fast oxidation process, following the typical hydrocarbon oxidation mechanism, with the formation of oxygen-containing groups, mainly hydroperoxides and carboxylic groups and to a minor extent ketones, esters, and lactones. In contrast, the single PE/PET layer showed excellent stability over all the treatment times.

We also observed that both yellowing and the monitoring of PP crystallinity are early indicators of oxidation. In particular, morphology changes induced a loss of mechanical properties, observable as embrittlement of the fibers of the layers. As a validation under natural conditions showed that the structural changes are the same as those obtained through artificial aging, we may suppose that long-term outdoor aging of PP-based surgical masks and filtering respirators disposed into the natural environment will result in fast and extensive oxidation, leading to their breakdown in the form of microfiber fragments, i.e., microplastics. Preliminary field observation confirmed our hypothesis [2,16,17]. Such partially oxidized PP microplastics with lower crystallinity would then enter the food web through the usual long-term process of bioassimilation.

## Figures and Tables

**Table 1:** Composition of the layers of selected face masks and respirator.

Selected Items	Layer Code	Composition	Fiber Ø (μm) ^1^
Surgical mask A	A1	spunbonded PP with blue dye	20–25
	A2	melt-blown PP	5–20
	A3	spunbonded PP	20–25
Surgical mask B	B1	spunbonded PP	20–25
	B2	melt-blown PP	5–20
	B3	spunbonded PP	20–25
Filtering respirator C	C1	spunbonded PP	20–25
	C2	PE/PET hot air cotton	10–20
	C3	spunbonded PP with light stabilizer	20–25
	C4	spunbonded PP with light stabilizer	20–25
	C5	spunbonded PP	20–25

^1^ By SEM measurements.

**Table 2:** The temperature of the main melting peak and χ_c_ of the PP layers as a function of the time of isothermal treatment at 110 °C. Values refer to the second scan.

	A1		A2		B1		C1		C3	
Time (h)	*T_m_* (°C)	χ_c_ (%)	*T_m_* (°C)	χ_c_ (%)	*T_m_* (°C)	χ_c_ (%)	*T_m_* (°C)	χ_c_ (%)	*T_m_* (°C)	χ_c_ (%)
0	165	46	158	46	164	45	164	46	156	47
100	163	50	156	50	165	45	-	-	-	-
250	141	45	157	48	165	44	165	46	154	47
500	140	42	156	48	165	44	141	41	154	47
1000	137	41	154	49	163	45	139	41	153	47

**Table 3:** The temperature of the main melting peak and χ_c_ of the PP layers as a function of the time of accelerated photoaging at 24 °C. Values refer to the second scan.

	A1		A2		B1		C1		C3	
Time (h)	*T_m_* (°C)	χ_c_ (%)	*T_m_* (°C)	χ_c_ (%)	*T_m_* (°C)	χ_c_ (%)	*T_m_* (°C)	χ_c_ (%)	*T_m_* (°C)	χ_c_ (%)
0	165	46	158	46	164	45	164	46	156	47
100	162	49	151	46	160	46	160	49	156	47
250	157	46	147	44	159	45	158	46	154	49
500	159	46	147	44	157	45	160	44	154	47
1000	158	45	- ^1^	- ^1^	158	45	160	45	154	47

^1^ No measurements were possible due to extensive embrittlement and pulverization of the sample.

## Data Availability

The data presented in this study are available on request from the corresponding author.

## Supplementary Materials

The following supporting information can be downloaded at: https://www.mdpi.com/article/10.3390/polym15041001/s1, Figure S1: ATR-FTIR spectra of layers C2 and C3; Figure S2: Optical micrograph of B1 and the dust formed from the extensive rupture of its fibers; Figure S3: DSC curves of A1 before and after 1000 h isothermal treatment; Table S1: Evolution of the CIELAB coordinates of PP layers as a function of the time of isothermal treatment at 110 °C; Table S2: Evolution of the CIELAB coordinates of PP layers as a function of the time of accelerated photoaging at 24 °C.
