# Supplementary material for: Study of the Long-Term Aging of Polypropylene-Made Disposable Surgical Masks and Filtering Facepiece Respirators
Source: Polymers (Basel). 2023 Feb 17;15(4):1001. doi: 10.3390/polym15041001 (PMC9962738; doi:10.3390/polym15041001)
Supplement: Supplementary file 1 [file polymers-15-01001-s001.zip › polymers-2193056-supplementary.pdf]

Study of the long-term aging of polypropylene-made disposable surgical masks and filtering facepiece respirators

Sandra Carreiras-Suárez [1,2], Lidia Domínguez-Ramos [1,2,3] and Massimo Lazzari [1,2,*]

[1] Departamento de Química Física, Facultade de Química, Universidade de Santiago de Compostela, Avenida das Ciencias s/n, 15782 Santiago de Compostela, Spain
[2] Centro Singular de Investigación en Química Biolóxica e Materiais Moleculares (CiQUS), Rúa Jenaro de La Fuente s/n, 15782, Santiago de Compostela, Spain
[3] Departamento de Ingeniería Química, ETSE, Universidade de Santiago de Compostela, Rua Lope Gómez de Marzoa s/n, 15782 Santiago de Compostela, Spain

* Correspondence: massimo.lazzari@usc.es

Supplementary Materials: Figure S1: ATR-FTIR spectra of layer C2 and C3; Figure S2: optical micrograph of B1 and the dust formed from the extensive rupture of its fibers; Figure S3: DSC curves of A1 before and after 1000 h isothermal treatment; Table S1: evolution of the CIELAB coordinates of PP layers as a function of the time of isothermal treatment at 110°C; Table S2: evolution of the CIELAB coordinates of PP layers as a function of the time of accelerated photoaging at 24°C.

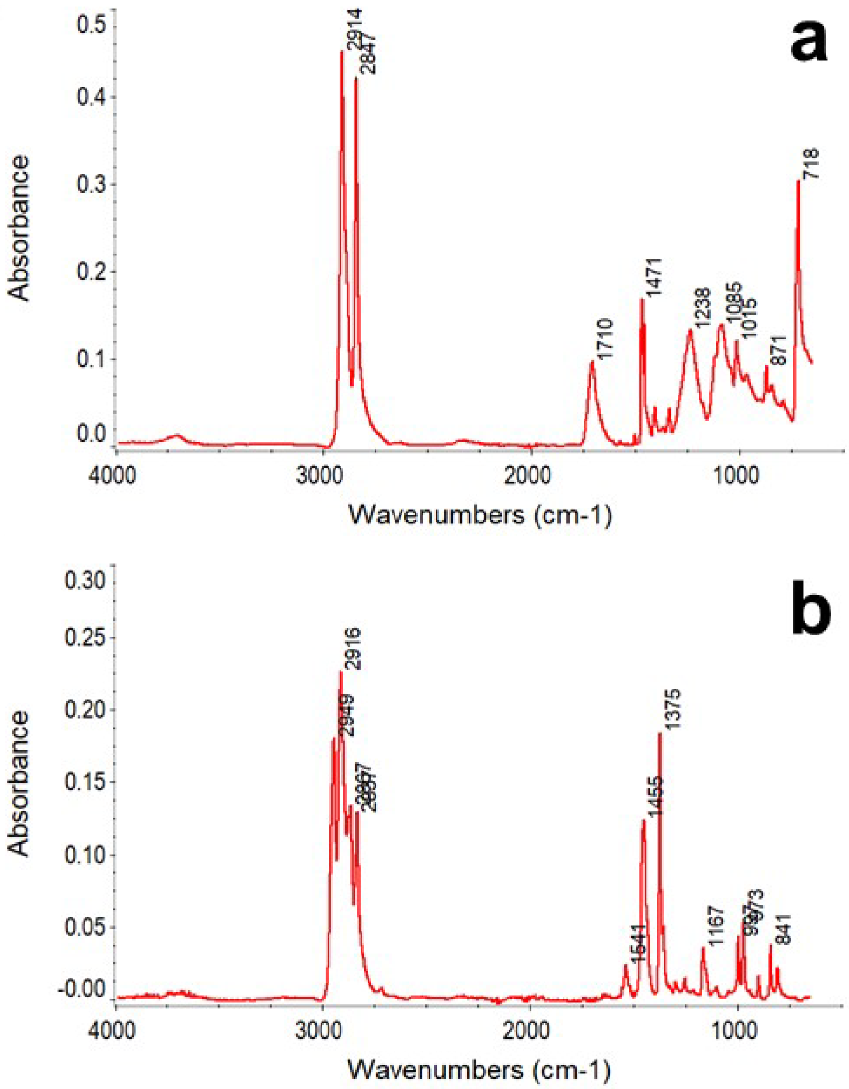

Figure S1. ATR-FTIR spectra of layer C2 (**a**) and C3 (**b**).

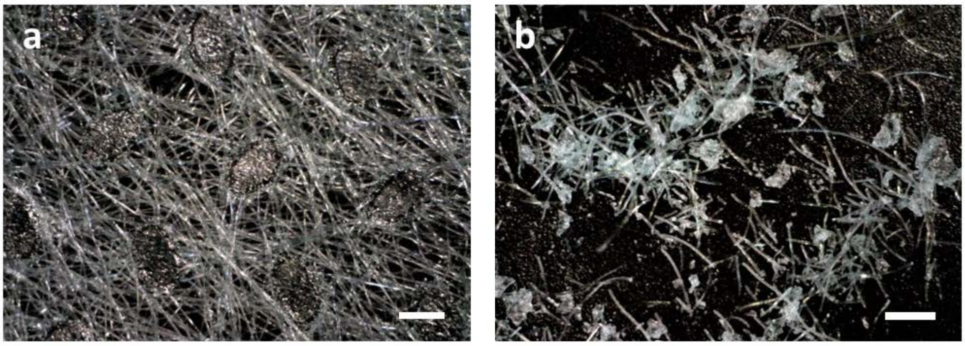

Figure S2. Optical micrograph of B1 (a) and the dust formed from the extensive rupture of its fibers (b). Scale bar 0.5 mm.

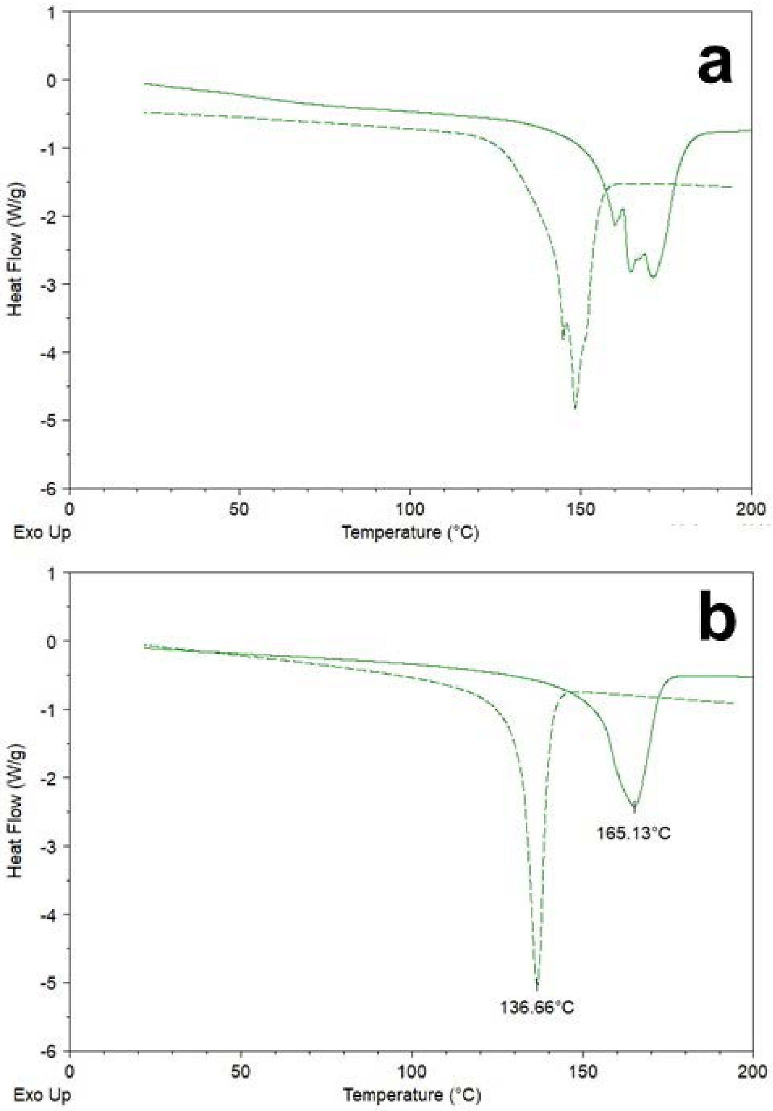

Figure S3. DSC curves of A1 before (solid line) and after 1000 h isothermal treatment (dashed line): first scan (**a**) and second scan (**b**).

Table S1. Evolution of the CIELAB coordinates of PP layers as a function of the time of isothermal treatment at 110°C.

	A1				A2				B1				C1				C3			
Time (h)	ΔL^*	Δa^*	Δb^*	ΔE	ΔL^*	Δa^*	Δb^*	ΔE	ΔL^*	Δa^*	Δb^*	ΔE	ΔL^*	Δa^*	Δb^*	ΔE	ΔL^*	Δa^*	Δb^*	ΔE
20	0.06	0.74	0.86	1.14	-0.63	0.25	0.25	0.72	-0.41	0.24	-0.24	0.53	-0.31	0.23	0.48	0.62	-0.30	0.22	0.21	0.43
100	-0.24	-0.45	-0.56	0.76	-0.04	0.05	0.95	0.95	0.41	0.05	0.75	0.86	-0.52	0.10	0.66	0.85	-0.07	-0.14	2.30	2.31
150	-0.01	0.46	-0.89	1.00	-0.06	-0.03	1.39	1.39	-0.32	0.03	0.29	0.43	-0.12	0.11	0.29	0.33	-0.29	-0.29	2.46	2.49
200	0.97	1.84	1.80	2.75	-0.40	-0.11	0.91	1.00	-0.11	-0.03	0.12	0.17	-0.20	0.05	0.33	0.39	-0.66	-0.57	2.43	2.58
250	-3.21	-5.02	-0.24	5.96	-0.39	-0.11	1.58	1.63	-0.45	0.02	-0.07	0.46	-0.02	0.05	0.39	0.39	-0.90	-0.38	2.40	2.59
325	-3.94	-6.07	2.21	7.57	-0.31	-0.36	1.63	1.70	-0.46	-0.09	0.22	0.52	-0.22	-0.05	0.34	0.41	-0.86	-0.34	2.38	2.55
500	-4.75	-8.08	4.35	10.33	-0.57	-0.37	1.80	1.92	-0.48	-0.14	0.10	0.51	-0.60	-0.81	3.06	3.22	-0.86	-0.32	2.58	2.74
750	-4.94	-8.58	6.39	11.78	-0.64	-0.40	1.25	1.46	-0.27	0.07	-0.17	0.33	-1.58	-3.24	14.10	14.55	-0.82	-0.41	2.50	2.66
875	-7.66	-13.93	7.49	17.57	-0.89	-0.27	1.20	1.52	-0.20	-0.05	0.66	0.69	-1.91	-3.04	14.23	14.68	-0.71	-0.39	3.02	3.13
1000	-13.38	-19.31	9.11	25.20	-0.36	-0.41	2.40	2.46	-0.40	-0.24	0.76	0.89	-1.51	-3.37	15.50	15.93	-0.81	-0.56	3.54	3.67

Table S2. Evolution of the CIELAB coordinates of PP layers as a function of the time as a function of the time of accelerated photoaging at 24°C.

	A1				A2				B1				C1				C3			
Time (h)	ΔL^*	Δa^*	Δb^*	ΔE	ΔL^*	Δa^*	Δb^*	ΔE	ΔL^*	Δa^*	Δb^*	ΔE	ΔL^*	Δa^*	Δb^*	ΔE	ΔL^*	Δa^*	Δb^*	ΔE
100	-0.60	-0.19	-0.34	0.72	-0.15	-0.07	0.31	0.35	-0.09	-0.11	0.27	0.31	0.20	-0.04	0.33	0.39	-0.36	-0.09	-0.01	0.37
250	-0.90	-0.12	-0.21	0.93	-0.08	-0.12	1.07	1.08	-0.33	-0.06	-0.05	0.34	-0.16	0.04	0.13	0.21	-0.34	-0.20	1.15	1.22
500	-3.52	0.97	0.23	3.66	- [1]	- [1]	- [1]	- [1]	-0.14	-1.48	0.10	1.49	-2.01	0.03	0.01	2.01	-0.89	-0.06	0.06	0.89
750	-10.36	2.44	2.03	10.84	- [1]	- [1]	- [1]	- [1]	1.21	-1.55	0.19	1.98	-3.87	0.07	0.37	3.89	-2.40	-0.13	-0.04	2.40
1000	-17.53	0.13	-1.41	17.59	- [1]	- [1]	- [1]	- [1]	-7.18	-1.56	-0.50	7.36	-5.44	-0.11	-0.51	5.46	-1.93	-0.02	-1.10	2.22

[1] no measurements were possible due to extensive embrittlement and pulverization of the sample.
